# Patterns of adverse childhood experiences and depressive symptom trajectories in young adults: A longitudinal study of college students in China

**DOI:** 10.3389/fpsyt.2022.918092

**Published:** 2022-07-25

**Authors:** Shuqin Li, Rui Wang, Erica Thomas, Zhicheng Jiang, Zhengge Jin, Ruoyu Li, Yan Qian, Xianbing Song, Ying Sun, Shichen Zhang, Ruoling Chen, Yuhui Wan

**Affiliations:** ^1^Department of Maternal, Child and Adolescent Health, School of Public Health, Anhui Medical University, Anhui, China; ^2^Anhui Provincial Key Laboratory of Population Health and Aristogenics, Anhui, China; ^3^Teaching Affairs Office, Anqing Medical College, Anhui, China; ^4^Faculty of Education, Health and Wellbeing, University of Wolverhampton, Wolverhampton, United Kingdom; ^5^Department of Human Anatomy, Histology and Embryology, Anhui Medical College, Anhui, China

**Keywords:** adverse childhood experiences, depressive symptom, latent class analysis, latent class growth model, college and university students

## Abstract

**Background:**

Adverse childhood experiences (ACEs) tend to cluster together in daily life, and most studies focus on the level of depression at certain points, but the dynamic process of depression is often neglected. Thus, research is urgently needed to explore the relationship between ACEs pattern and trajectory of depressive symptom levels at multiple time points in order to provides early targeted interventions to those who are most at risk.

**Objective:**

We aimed to explore patterns of ACEs, including types and timing, associated with depression trajectories in college students.

**Methods:**

A school-based health survey was used to collect data as part of a longitudinal study in two medical college in Anhui province, China. Questionnaires were issued to 3,662 participants aged 17–22 and recorded details of ACEs (types and timing) and depression. Latent class analysis (LCA) was used to identify “patterns” of ACEs type and timing. Depressive symptom trajectories employed latent class growth analysis (LCGA). Multiple logistic regressions were employed to evaluate the relationships between ACEs patterns and depressive symptom trajectories.

**Results:**

We identified five ACEs patterns: “High neglect/emotional abuse/community violence,” “High neglect/emotional abuse,” “High neglect/family dysfunction,” “High neglect,” “Low ACEs.” We traced three depression trajectories: “High depressive symptom” “Moderate depressive symptom,” “Low depressive symptom.” “High neglect/emotional abuse/community violence,” “High neglect/emotional abuse” and “High neglect/family dysfunction” demonstrated a high risk for “High depressive symptom” and “Moderate depressive symptom.” “High neglect” showed a high risk for “Moderate depressive symptom” but not for “High depressive symptom” (*P* < 0.05).

**Conclusions:**

The findings address the need for a comprehensive consideration of exposure to childhood adversity associated with the risk of depression in young adults through identifying more problematic ACEs patterns amongst exposed children.

## Introduction

Adverse childhood experiences (ACEs) are highly stressful and/or traumatic events or situations that occur during childhood or adolescence. ACEs can include physical, sexual or emotional abuse, neglect, exposure to domestic violence, alcohol or drug abuse, divorce or separation of parents and family financial difficulties (1). Exposure to childhood adversity is not uncommon, 57% of people reporting at least one ACEs and 13% reporting at least four (2).

ACEs can have long term negative consequences for psychosocial functioning and risk behaviors and increase the likelihood of both physical and mental health problems later in life. A reviewer summarized that the history of ACEs can result in complex clinical profiles with several co-occurring mental and somatic disorders such as posttraumatic stress disorder, depression, borderline personality disorder, obesity and diabetes in adulthood (3). Bellis et al. illustrated that ACEs were attributed to about 30% of cases of anxiety and 40% of cases of depression in north America and more than a quarter of both conditions in Europe (4). The type of childhood adversity experienced also has a differential impact on health outcomes and risk behaviors. Trotta et al. found evidence of a significant association between parental separation in childhood and longer admissions to psychiatric wards during 1-year follow-up and 2-fold increased odds of non-compliance with medication compared to those not separated from their parents (5). Beal et al. suggest that family violence was associated with poorer psychological wellbeing and quality of life, while family instability was associated with cigarette and marijuana use (6). Schalinski et al. explored effects of childhood adversities on psychotic disorders and found emotional and physical neglect were particularly related to psychotic disorders symptom severity (7). Recent evidence indicates an additional impact of timing of ACEs on health outcomes in adulthood, suggesting that there may be a stress sensitive period in a child's brain development (8, 9). It has been suggested that during certain vulnerable developmental phases the risk for subsequent ACE-related disorders is increased (3). ACEs do not occur in isolation, instead, childhood adversities tend to cluster together. Thus the estimate of a single adverse experience cannot accurately reflect the actual exposure in daily life (10, 11). Person-centered approaches, such as latent class analysis (LCA), represent a conceptual and analytical shift from the more commonly used ACEs score (12, 13). LCA can identifies homogenous population subgroups with similar constellations of ACEs risk (14).

Understanding the link between ACEs and mental health among young adults is critical given the specificity during developmental period since college students are in the stage of transition from adolescence to adulthood and their psychology, physiology and surrounding environment have undergone major changes (15). Consequences emerging in early adulthood, these issues may extend or augment the influences throughout individuals' lifetimes if no proper understanding is reached and interventions made at the issues' onset. Consistent findings highlight ACEs are non-negligible factor of poor mental health in adolescents (16, 17). For example, Wan et al. illustrated non-suicidal self-injury, suicidal ideation and suicide attemptwere significantly associated with increased ACEs in community adolescent populations (16). Emerging research also demonstrates ACEs during childhood contributes to health consequences in later adulthood (18–20). Among college students, studies have demonstrated that ACEs is associated with risk behaviors, and mental health (21, 22). Specifically in China, ACEs were prevalent among Chinese young adults and had deleterious effects on their psychological wellbeing (23–25). For instence, Wang et al. found that Childhood trauma was positively correlated with indifference, loneliness, and suicidal ideation, and negatively correlated with identification with all humanity in college students (23).

There is evidence that ACEs significantly increase the risk of depression in adulthood (26). Most studies however, focus on the level of depression at certain points, but the dynamic process of depression is often neglected. Thus, research is urgently needed to explore the change trajectory of depressive symptom levels at multiple time points in relation to ACEs. This would not only better reflect the actual development of depressive symptom, but more precisely predict the severity of the problem in the future (27).

To date however, these studies indicate that ACEs are important to the mental health of college students, but few studies have focused on the ways in examinedg and compareding the relative effects of ACEs patterns combined with timing on the developmental trajectories of depressive symptom in adult. This study aimed to (1) use Latent Class Analysis (LCA) to explore patterns of ACEs combined with type and timing in college students; (2) employ a Latent Class Growth Model (LCGM) to distinguish depressive symptom trajectories in a longitudinal study; (3) analyse the relationships between ACEs patterns and depressive symptom trajectories in Chinese students.

## Materials and methods

### Sample and procedure

The present study used data from a longitudinal study of students in two medical college in Anhui province, China. In each school, a cluster sampling method was used to extract all students from each major A total of 4,211 students (1,137 males; 3,074 females) from grade 1, were interviewed in the baseline study in November 2019 (T1). Their ages ranged from 17 to 22, average age was 19.2 (SD = 1.0) years. Three thousand, eight hundred and nineteen students (90.61%; 2,787 females, 1,032 males) completed the follow-up survey 6 months later (T2). Three thousand, six hundred and sixty two students (86.98%; 976 males; 2,686 females) completed the follow-up survey 12 months post baseline (T3). There was no statistical significance in gender difference between lost to follow-up and not lost to follow-up (χ^2^ = 2.014, *P* = 0.156). The design and data collection procedures were approved by the Ethics Committee of Anhui Medical University (20170290). Informed consent was obtained from participants.

### Measures

#### Adverse childhood experiences

ACEs were defined as experiences during childhood of maltreatment and/or household dysfunction, peer bullying and community violence (28). Child maltreatment was evaluated using the Chinese version of the Childhood Trauma Questionnaire- Short Form (CTQ-SF) (29) originally developed by Bernstein et al. (30). The Chinese version of the CTQ-SF has 28 items (including three validity items) assessing five domains of childhood trauma: physical abuse, sexual abuse, emotional abuse, physical neglect and emotional neglect. Each domain contains 5 items. Participants were asked about any abusive childhood experiences that occurred before the age of 18. Response scores ranged from 1 = “never,” 2 = “rarely,” 3 = “sometimes,” 4 = “often,” to 5 = “very often.” Respondents were defined as “exposed to a category” if they responded “rarely” “very often,” “often,” or “sometimes” to any item in that category. Taking into account the high correlation between physical neglect and emotional neglect in this study, we combined these two domains into neglect. The Cronbach's α coefficient for the CTQ-SF was 0.740.

Household dysfunction items were derived from Felitti et al. (31) adapted to the reality in China Household dysfunction was assessed using four items: (1) divorce/separation of parents, (2) witnessed domestic violence, (3) having lived with someone with alcohol or gambling problems, and (4) depressive symptom or mental illness. Respondents were defined as having been exposed to household dysfunction if they responded “yes” to any of these items.

Peer bullying and community violence items were derived from the Adverse Childhood Experiences International Questionnaire (ACE-IQ) (1). Peer bullying was assessed using two items: (1) How often were you hit, kicked, pushed, shoved around, or locked indoors? and (2) How often were you left out of activities on purpose or completely ignored? and Community violence was assessed using two items (1) Did you see or hear someone being beaten up in real life? (2) Did you see or hear someone being threatened with a knife or gun in real life? Response scores for all items ranged from 1 = “never,” 2 = “rarely,” 3 = “sometimes,” 4 = “often,” to 5 = “very often.” Respondents were defined as “exposed to a category” if they responded “rarely” “very often,” “often,” or “sometimes” to any item in that category.

Additionally, the timing of exposure to each ACE item was investigated. In the data analysis, timing of exposure to ACEs was grouped into (1) before middle school, (2) in middle school, (3) in high school.

#### Depressive symptom

Depressive symptom was evaluated using the Zung Self-Rating Depression Scale (SDS). The SDS is a 20-item questionnaire widely used to quantify the severity of affective, somatic, psychomotor, and psychological depressive symptoms (32, 33). Response scores range from 1 = “a little of the time,” 2 = “some of the time,” 3 = “good part of the time,” 4 = “most of the time” for each item. Total scores rang from 20 to 80 with higher scores indicating more severe depressive symptoms. The composite SDS score was used in the present study. The Cronbach's α coefficient for the SDS were 0.797 (in T1), 0.862 (in T2), 0.858 (in T3), respectively.

### Covariates

In the baseline survey, we collected data on sociodemographics, including age, gender, urban/rural, parents' education level (less than junior middle school, junior middle school, senior middle school, college or higher), self-perceived economic status of the family (good, moderate, poor), only child (yes or no), and smoking and drinking in the previous month (yes or no), which might affect the associations between ACEs and mental health outcomes (34, 35). In follow-up at T2 and T3, we also collected data on and smoking and drinking in the previous month.

### Statistical analysis

We analyzed data of 3,662 participants who completed all three interviews from T1 to T3. To reduce the risk of bias from missing data, we conducted multiple imputation in SPSS for all participants at T1, T2, T3, respectively. Continuous variables were described using mean and standard deviation (SD), while categorical variables were described using frequency counts and percentages. Differences between depressive symptom trajectories were assessed using the χ^2^ test for categorical variables.

In order to identify clusters of ACEs, latent class analysis (LCA) was used to identify homogeneous, mutually exclusive “patterns” of ACEs type and timing.The 1-class solution was tested first and then the number of classes was incrementally increased until the best fitting model was identified. The ACEs classes were determined based on model fit indices: Akaike's Information Criteria (AIC) (36), Bayesian Information Criteria (BIC) (37) and sample size adjusted BIC (ssaBIC) (38), entropy, and *p*-value for Lo-Mendell-Rubin Test (LMRT) (39) and Bootstrapped Likelihood Ratio Test (BLRT) (40). Multiple sets of random starting values would be specified for each tested LCA model. Individuals were assigned to latent classes based on the largest posterior class membership probabilities that could be obtained from their observed responses and the estimated parameters of the LCA model. The best model was identified according to substantive interpretation and multiple fit statistics. After the determination of the best fitting model, the association between a set of demographic covariates and class membership was evaluated using the modified three-step procedures (R3STEP auxiliary command) recommended by Vermunt (41) to ensure the minimal bias of the effects of the covariates (gender, urban/rural, parents' education level, self-perceived economic status of the family, only child) on the classes. This approach took into account measurement error associated with the most likely class membership, while preserving the stability of class formation. Sensitivity analyses using “often” “very often” as “exposed to a category” to any item to classify ACEs pattrens.

Depressive symptom trajectories employed latent class growth analysis (LCGA). Like LCA, the 1-class solution was tested first and then the number of classes were incrementally increased until the best fitting model was identified. The depressive symptom trajectories were determined based on model fit indices like AIC, BIC, ssaBIC, entropy, LMRT and BLRT.

Multiple logistic regressions were employed to evaluate the relationships between ACEs patterns and depressive symptom trajectories as odds ratios (ORs) and adjusted odds ratios (aORs) with 95% confidence intervals (CIs). In the multiple logistic regressions, we adjusted for age, gender, urban/rurality, parents' education level, economic status of family, and only child. LCA and LCGA were conducted using Mplus 7.4 and other analyses were conducted using SPSS software, Windows version 23.0.

## Results

### Patterns of ACEs

Models of ACE patterns with one to nine classes were tested in the LCA (Supplementary Table DS 1). The 5-class model was selected based on the lower BIC, ssaBIC and higher entropy (0.833), and the average posterior class membership probability scores were acceptable among groups (0.843–0.856; Supplementary Table DS 2).

**Figure 1:**
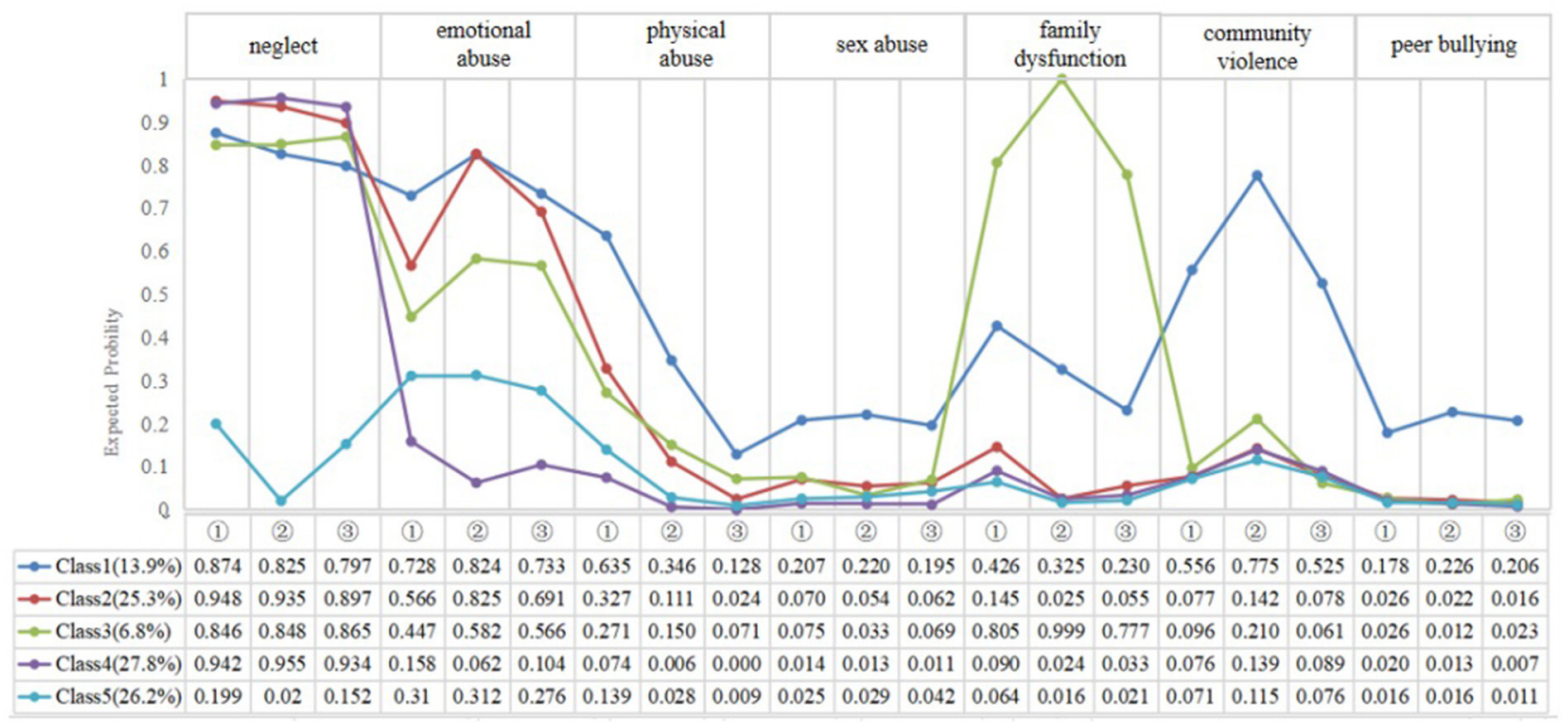
Plot of 5 latent classes of adverse childhood experiences. (① = Before middle school; ② = In middle school; ③ = In high school).

Figure 1 shows the 5-class model of ACEs and item-response probabilities for the seven types and three stages of ACEs for each class. Class 1, characterized by a high probability of exposure to neglect, emotional abuse and community violence combined with type and timing was labeled as “High neglect/emotional abuse/community violence” (13.9%); Class 2, comprised of individuals with high probabilities of exposure to emotional abuse and neglect, was labeled as “High neglect/emotional abuse” (25.3%); Class 3, consisting of individuals likely to report family dysfunction, was labeled as “High neglect/family dysfunction” (6.8%); Class 4, made up of individuals with a high probability of exposure to neglect, was labeled as “High neglect” (27.8%); Class 5, characterized by a low probability of exposure to each of the ACEs, was labeled as “Low ACEs” (26.2%). Sensitivity analyses also shows the 5-class model of ACEs was the better model for the seven types and three stages of ACEs for each class (Supplementary Table DS 3; Supplementary Figure DS 1).

### Depressive symptom trajectories

Models of depression trajectories with one to four classes were tested in the LCGA (Supplementary Table DS 4). The 3-class model was selected based on the lower BIC, ssaBIC and valid entropy (0.806), and the average posterior class membership probability scores were acceptable among groups (0.843–0.856; Supplementary Table DS 5).

**Figure 2:**
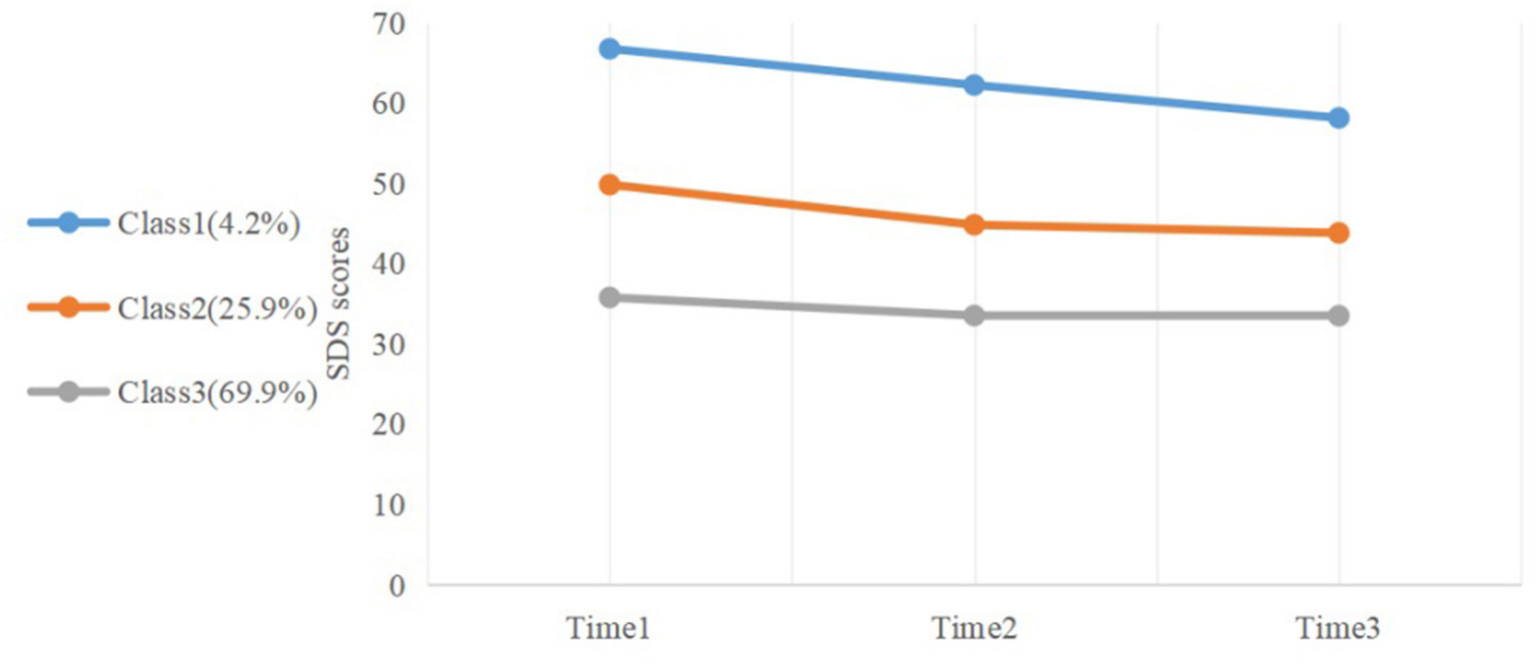
Plot of depressive symptom trajectories during college, derived from latent class growth analysis (LCGA).

Figure 2 shows the course trajectories of the three latent classes identified. Class 1 with initial depressive symptom severity higher than other classes was labeled as “High depressive symptom” (4.2%); class 2 with a slower remission course trajectory of moderate depressive symptom severity was labeled as “Moderate depressive symptom” (25.9%), and class 3 showing a chronic course trajectory of lower depressive symptom severity was labeled as “Low depressive symptom” (69.9%).

### Characteristics of participants

The average age of participants at baseline was 19.2 (±1.0) years, 73.3% female and 26.7% male. Table 1 shows the characteristics of all potential concomitants of depressive symptom. Considering each explanatory variable separately, economic status of family, drinking, smoking at T3 and ACEs patterns were found to have a significant association with depressive symptom trajectories (*P* < 0.05). There was no significant association between gender, urban/rural, parents' education level, and only one child, with depressive symptom trajectories.

**Table 1:** Characteristics of participants by depressive symptom trajectories, data shown as *n* (%).

**Variables**	**High depressive symptom**	**Moderate depressive symptom**	**Low depressive symptom**	***χ^2^*** **/t**	***P***
Genders				3.667	0.160
Males	39 (26.4)	255 (29.1)	682 (25.8)		
Females	109 (73.6)	620 (70.9)	1957 (73.3)		
Urban/rurality				0.172	0.918
Rurality	96 (64.9)	556 (63.5)	1669 (63.2)		
Urban	52 (35.1)	319 (36.5)	970 (36.8)		
Father's education level				2.685	0.847
College or more	12 (8.1)	62 (7.1)	205 (7.8)		
Senior middle school	26 (17.6)	143 (16.3)	435 (16.5)		
Junior middle school	60 (40.5)	408 (46.6)	1227 (46.5)		
Less than junior middle school	50 (33.8)	262 (29.9)	772 (29.3)		
Mother's education level				9.735	0.136
College or more	5 (3.4)	45 (5.1)	114 (4.3)		
Senior middle school	14 (9.5)	85 (9.7)	308 (11.7)		
Junior middle school	41 (27.7)	317 (36.2)	903 (34.2)		
Less than junior middle school	88 (59.5)	428 (48.9)	1314 (49.8)		
Economic status of family				63.928	<0.001
Good	3 (2.0)	30 (3.4)	88 (3.3)		
Moderate	59 (39.9)	495 (56.5)	1735 (65.7)		
Poor	86 (58.1)	350 (40.0)	816 (30.9)		
Only child					
Yes	39 (26.4)	194 (22.2)	568 (21.5)	1.971	0.373
No	109 (73.6)	681 (77.8)	2071 (78.5)		
Smoking at T1 (Yes)	9 (6.1)	58 (6.6)	143 (5.4)	1.814	0.404
Drinking at T1 (Yes)	31 (20.9)	172 (19.7)	419 (15.9)	8.375	0.015
Smoking at T2 (Yes)	9 (6.1)	34 (3.9)	81 (3.1)	4.763	0.092
Drinking at T2 (Yes)	32 (21.6)	155 (17.7)	329 (12.5)	22.174	<0.001
Smoking at T3 (Yes)	14 (8.8)	45 (5.1)	94 (3.6)	12.449	0.002
Drinking at T3 (Yes)	26 (17.6)	96 (11.0)	209 (7.9)	21.089	<0.001
ACEs patterns					
High neglect/emotional abuse/community violence	70 (47.3)	184 (21.0)	237 (9.0)	387.615	<0.001
High neglect/emotional abuse	35 (23.6)	301 (34.3)	606 (23.0)		
High neglect/family dysfunction	14 (9.5)	78 (8.9)	162 (6.1)		
High neglect	15 (10.1)	206 (23.5)	790 (29.9)		
Low ACEs	14 (9.5)	106 (12.1)	844 (32.0)		

*ACEs, adverse childhood experiences*.

### Types and timing of ACEs with depressive symptom trajectories

Table 2 shows the relationship between type of ACEs and timing with depressive symptom trajectories. If the reference category was “Low depressive symptom,” emotional abuse in high school, sexual abuse in middle school and peer bullying, demonstrated a higher rate of risk for “High depressive symptom” than their counterparts. Moreover, the findings indicate that neglect, emotional abuse and peer bullying comparably increased the risk (~1–3 times higher) of “Moderate depressive symptom” in contrast to their counterparts (*P* <0.05).

**Table 2:** Logistic regression models of depressive symptom trajectories by ACEs types and timing (Reference: No ACEs).

**Type**	**Timing**	***n***	**High depressive symptom** ^a^		**Moderate depressive symptom** ^a^		**High depressive symptom** ^b^	
			**OR (95% CI)**	***P***	**OR (95% CI)**	***P***	**OR (95% CI)**	***P***
Neglect	Before middle school	2,684	1.113 (0.580–2.134)	0.748	1.516 (1.154–1.993)	0.003	0.734 (0.375–1.436)	0.366
	In middle school	2,490	1.852 (0.922–3.722)	0.084	1.410 (1.066–1.865)	0.016	1.314 (0.642–2.687)	0.455
	In high school	2,551	1.749 (0.928–3.294)	0.084	1.347 (1.039–1.745)	0.024	1.298 (0.680–2.480)	0.429
Emotional abuse	Before middle school	1,466	1.150 (0.761–1.738)	0.506	1.244 (1.036–1.494)	0.020	0.925 (0.608–1.407)	0.715
	In middle school	1693	1.651 (1.022–2.667)	0.040	1.310 (1.071–1.603)	0.009	1.260 (0.773–2.054)	0.354
	In high school	1,526	2.143 (1.368–3.358)	0.001	1.477 (1.219–1.788)	<0.001	1.451 (0.918–2.294)	0.111
Physical abuse	Before middle school	903	0.852 (0.547–1.325)	0.476	0.912 (0.740–1.125)	0.390	0.933 (0.598–1.457)	0.762
	In middle school	350	1.258 (0.737–2.148)	0.400	1.034 (0.763–1.403)	0.827	1.216 (0.715–2.070)	0.471
	In high school	114	1.026 (0.479–2.195)	0.948	0.884 (0.546–1.431)	0.616	1.161 (0.555–2.428)	0.692
Sex abuse	Before middle school	226	1.215 (0.664–2.224)	0.523	1.447 (1.046–2.001)	0.026	0.840 (0.466–1.514)	0.562
	In middle school	211	4.1221 (2.244–7.568)	<0.001	1.181 (0.810–1.723)	0.386	3.488 (1.904–6.388)	<0.001
	In high school	226	0.628 (0.302–1.308)	0.214	1.269 (0.891–1.807)	0.186	0.495 (0.240–1.019)	0.056
Family dysfunction	Before middle school	706	1.233 (0.770–1.973)	0.384	1.334 (1.063–1.675)	0.013	0.924 (0.576–1.482)	0.743
	In middle school	478	1.569 (0.899–2.736)	0.113	0.878 (0.645–1.196)	0.409	1.787 (1.023–3.121)	0.041
	In high school	416	1.019 (0.576–1.801)	0.949	1.234 (0.914–1.666)	0.170	0.826 (0.468–1.456)	0.508
Community violence	Before middle school	524	1.430 (0.888–2.303)	0.141	1.293 (1.010–1.654)	0.041	1.106 (0.689–1.776)	0.676
	In middle school	830	1.169 (0.729–1.873)	0.517	1.034 (0.821–1.303)	0.775	1.130 (0.702–1.818)	0.615
	In high school	519	1.614 (0.982–2.562)	0.059	1.347 (1.044–1.738)	0.022	1.198 (0.729–1.969)	0.476
Peer bully	Before middle school	156	2.777 (1.481–5.204)	0.001	1.608 (1.066–2.427)	0.024	1.726 (0.952–3.131)	0.072
	In middle school	167	3.286 (1.783–6.057)	<0.001	2.055 (1.364–3.097)	0.001	1.599 (0.898–2.849)	0.111
	In high school	144	3.497 (0.839–6.648)	<0.001	2.478 (1.585–3.873)	<0.001	1.411 (0.788–2.528)	0.247

*ACEs, Adverse Childhood Experiences; Adjusted for age, gender, parent' education level, economic status of family, urban/rurality, only child, smoking and drinking;*

a *Low depression was used as the reference category;*

b *Moderate depression was used as the reference category*.

### Patterns of ACEs with depressive symptom trajectories

In Table 3, associations between ACEs and depressive symptom trajectories are presented. If the reference category was “Low depressive symptom,” “High neglect/emotional abuse/community violence,” “High neglect/emotional abuse” and “High neglect/family dysfunction” demonstrated a higher rate of risk for both moderate and high depressive symptom trajectories than “Low ACEs” (*P* < 0.001). “High neglect” showed a high rate of risk for “Moderate depressive symptom” but not for “High depressive symptom.” If the reference category was “Moderate depressive symptom,” only “High neglect/emotional abuse/community violence” demonstrated a higher rate of risk for “High depressive symptom” than “Low ACEs” (*P* < 0.05). The strongest association with “High depressive symptom” and “Moderate depressive symptom” could be observed in “High neglect/emotional abuse/community violence” patterns (*P* < 0.05).

**Table 3:** Logistic regression models of depressive symptom trajectories by ACEs types and timing (Reference: Low ACEs).

**ACEs patterns**	***N*** **(%)**	**High depressive** **symptom**^a^^*^		**Moderate depressive** **symptom**^a^^*^		**High depressive symptom** ^b^ ^*^		**High depressive** **symptom**^a^^#^		**Moderate depressive** **symptom**^a^^#^		**High depressive** **symptom**^b^^#^	
		**OR (95%CI)**	***P***	**OR (95%CI)**	***P***	**OR (95%CI)**	***P***	**OR (95%CI)**	***P***	**OR (95%CI)**	***P***	**OR (95%CI)**	***P***
High neglect/emotional abuse/community violence	509 (13.9)	17.806 (9.854–32.174)	<0.001	6.182 (4.676–8.172)	<0.001	2.880 (1.547–5.363)	0.001	14.199 (7.719–26.117)	<0.001	5.510 (4.141–7.331)	<0.001	2.577 (1.363–4.872)	0.004
High neglect/emotional abuse	926 (25.3)	3.482 (1.857–6.528)	<0.001	3.955 (3.096–5.051)	<0.001	0.880 (0.456–1.700)	0.704	2.849 (1.508–5.383)	0.001	3.731 (2.915–4.777)	<0.001	0.764 (0.393–1.483)	0.426
High neglect/family dysfunction	249 (6.8)	5.210 (2.437–11.136)	<0.001	3.834 (2.736–5.371)	<0.001	1.359 (0.613–3.014)	0.450	3.803 (1.749–8.271)	0.001	3.526 (2.504–4.965)	<0.001	1.079 (0.480–2.424)	0.855
High neglect	1,018 (27.8)	1.145 (0.549–2.387)	0.719	2.076 (1.611–2.675)	<0.001	0.551 (0.257–1.185)	0.127	0.985 (0.468–2.075)	0.968	1.970 (1.523–2.547)	<0.001	0.500 (0.231–1.083)	0.079
Low ACEs	959 (26.2)	1		1		1		1		1		1	

*ACEs, Adverse Childhood Experiences;*

a *Low depressive symptom was used as the reference category*

b *Moderate depressive symptom was used as the reference category*.

* *Crude;*

# *Adjusted for age,gender, parent' education level, economic status of family, urban/rurality, only child, smoking and drinking*.

## Discussion

In this large-scale cohort study, we analyzed the association between types and timing of ACEs and depressive symptom trajectories and found a significant association between childhood neglect, emotional abuse, sexual abuse and peer bullying with depressive symptom in young adults. The analysis also showed that emotional abuse in high school and sexual abuse in middle school brought about increased risk of depressive symptom. The impact of ACEs types and timing on depressive symptom remains controversial in previous studies. Previous research illustrated that various types of ACEs resulted in different status of mental health. Literature pointed to the link between sibling bully-victim, emotional neglect, emotional abuse and depressive symptom (42–44). Schalinski et al. believed that ACEs type and timing specific effects had stronger prediction for depression symptoms, and their findings showed that the emotional neglect at age 8–9 aggravated depression, yet other types had weaker association with depression symptoms like physical maltreatment, physical neglect and time (43). Since ACEs are multi-faceted (e.g., different types, timing of exposure), the estimate of a single component of childhood adversity may not fully reflect the actual exposure in daily life. In other words, the complexity and multidimensionality of ACEs call for advanced statistical techniques to improve the assessment.

The LCA analysis allowed for new insights into the differentiated risk of specific ACEs patterns for health outcomes amongst college students in China. Five patterns of ACEs exposure were identified *via* the application of LCA in this cohort study: High neglect/emotional abuse/community violence, High neglect/emotional abuse, High neglect/family dysfunction, High neglect, and Low ACEs. Moreover, apart from the investigation of the patterns of ACEs types, the timing of ACEs exposure patterns was another focus of our study. We found in five patterns the exposure to each type ACEs before 18 years old was not in a single stage but a durative course instead, implying a steady trend that could be seen among participants with ACEs experiences. This differs from what previous researchers have found. For example, a study with 9,310 individuals in the United States identified five latent classes: no/low abuse (58.5%), child physical abuse (15.6%), adolescent emotional abuse (8.8%), child and adolescent physical and emotional abuse (16.0%), and child and adolescent sexual abuse (1%) (45). Similarly, a study of 674 children aged 10–12 clarified four patterns: non-maltreated (48.0%), chronic, multi-subtype (30.0%), neglect only (16.0%), single subtype (6.0%) (46). In view of the above findings, ACEs patterns not only have particular exposure of types in each class but also have difference in developmental timing. Nevertheless, our study points to a continuous development of ACEs, which may be in line with the actual situation of China, since Chinese parenting style maintains high stability before children's entrance to college (47). Likewise, different samples, definitions of ACEs and control variables should be considered in the interpretation of the results of this study.

As illustrated in previous studies (45, 48, 49), different exposure patterns of ACEs could lead to various kinds of health problem. Lee et al. (48) identified four patterns in a National Longitudinal Study of Adolescent and Adult Health (Add Health), showing that “Child maltreatment” increased the risk of depression, anxiety, and PTSD while the “Community violence” class was liable to reported PTSD. Similarly, based on a study with 1346 university students in East Asia, students in the “Household violence” class reported significantly higher depression and maladjustment symptoms, and both the “Household violence” class and the “Household dysfunction” class had a higher risk of anxiety symptoms (49). Ziobrowski et al. (45) found that women and men in the “Adolescent emotional abuse” class, “Child and adolescent physical and emotional abuse” class, and women in “Child and adolescent sexual abuse” class had a greater prevalence of high depressive symptoms than those in “No/low abuse” class. In the current study, latent ACEs classes were found to be uniquely linked with depression, providing support for the validity of the identified classes, which may capture varying levels of risk associated with different patterns of ACEs. Our findings traced the differences in mental health in young adulthood by ACEs patterns, indicating that future research on young adults' mental health should focus on types and timing of ACEs.

In line with previous studies, we found that individuals with “High neglect/emotional abuse/community violence” suffered higher rates of high depressive symptom trajectory. A study in Germany involving diagnostic interviews with 311 depressed treatment-seeking outpatients reported that patients in the “Severe abuse and neglect” class had a significantly higher likelihood for comorbidities in depression (11). Kim et al. (50) found that the “High Adversity” and “Child Abuse” classes were prone to major depression symptoms compared to the “Low Adversity” class in the National Epidemiologic Survey on Alcohol and Related Conditions III in America. Current study prompted that neglect, emotional abuse, community violence and family dysfunction likely occur in childhood of which neglect should be focused on particularly since a most likely co-occurrence could be observed between neglect and other forms of ACEs. In addition, we identified a distinct class of participants who suffered predominantly from “High neglect,” showing a high rate of risk for “Moderate depressive symptom” rather than “High depressive symptom.” Previous study found that higher neglect severity was associated with smaller bilateral amygdala volume and bilateral hippocampal volume across traumatized individuals (51). This finding reminded the researchers that high neglect may not lead to high levels of depressive symptom, but more likely to cause mild-to-moderate levels of depressive symptom, a point which needs to be heeded for prevention. Several lines of evidence suggest that type and timing specific effects showed stronger prediction for symptoms depression. For example Schalinski et al. found that the emotional neglect at age 8–9 enhanced symptoms of depression (43). However, in our study, each type of ACEs was exposed in a durative process and indicated that student depressive symptoms might be explained by chronic exposure to ACEs. Similarly, Dunn found that child psychopathology symptoms were largely explained by the accumulation and recency of exposure to adversity, rather than sensitive periods (52).

Furthermore, current study also indicated that economic status of family, drinking and smoking were found to have a significant association with depressive symptom trajectories. Previous studies have shown that depression and substance use disorders are highly prevalent in the general population and often co-occur within the same individual (53, 54). Some people with any mental illness also have a substance use disorder. Conversely, those who abuse alcohol and other drugs also have a mental illness (55–57). Studies illustrated that depression and substance use disorder interact with each other, and ACEs are a risk factor for both depression and substance use disorder (58). In our study, there was significant association between ACEs and depressive symptoms after adjusting for somking and drinking.

### Strengths and limitations

A Person-centered approach was used to identify distinct patterns of ACEs combined with type and timing; this is a more concise representation of ACE subtypes compared to previous methods. Several subtypes of ACE were examined in this study, including neglect, abuse, family dysfunction, bullying and community violence, lending itself to a more comprehensive analysis. Additionally, previous studies have measured depressive symptom at a single time point. This study used LCGM to analyse depressive symptom trajectories at three time points over a 12 month period, thus recognizing and accounting for the dynamic process of depressive symptom.

Several limitations of this study should not be overlooked. First, the use of self-reported questionnaires for data collection purposes cannot rule out the possibility of recall bias, which may influence the strength of the observed relationships but recent work suggests that recall bias does not explain associations between retrospective reports of childhood adversity and psychopathology (59). Second, the analysis of this study centers on the patterns of ACEs types and timing, falling short of the mixed influence of other factors like frequency of ACEs and ACEs perpetrators, both of which have been found to influence health outcomes to varying extents (60). Third, our study included students from two medical universities in China. Caution should be taken when generalizing the findings of this study to other population groups. More research is required to investigate distinct patterns of ACEs combined with type and timing in the general population of young adults, middle aged and older adults in China. Fourth, depressive symptom trajectories were determined by measurements taken at three time points. Calls have been made to increase the frequency of measurements to ensure greater accuracy (27). Finally, it should be noted that ACEs affect people differently, thus the accuracy of participant classification based on posterior probabilities may vary from person to person. Person-to-person variation may distort estimates in subsequent analyses of classes, especially if individual differences remain to be overlooked.

## Conclusion

ACEs have latent individual heterogeneity among college students in China. The current study enhances our understanding of the relationship between patterns of ACEs, types of ACEs and timing with depressive symptom trajectories. These findings address the need for a comprehensive consideration of ACEs exposure associated with the risk of depressive symptom in young adults by identifying more problematic ACEs patterns amongst exposed children. This study illustrates a novel approach to the measurement of childhood adversity in order to identify populations at risk of depressive symptom later in life. This provides a unique opportunity for service providers to deliver early targeted interventions to those who are most at risk.

## Data availability statement

The raw data supporting the conclusions of this article will be made available by the authors, without undue reservation.

## Ethics statement

The studies involving human participants were reviewed and approved by Ethics Committee of Anhui Medical University (20170290). The patients/participants provided their written informed consent to participate in this study.

## Author contributions

SL and RW reviewed the topic related literature and drafted the first version of manuscript. SL, RW, ZJia, ZJin, RL, YQ, and XS performed the study design, coordination, and data collection. SL and YW worked on data analysis. ET, YS, SZ, and RC involved in interpretation of the data and revision of the manuscript. YW performed the study design, carried out study supervision, revision of the manuscript, and is the guarantor for the study. All authors checked interpreted results and approved the final version.

## Funding

Funding for the project was provided by National Natural Science Foundation of China (82073576) and the Natural Science Research of Universities in Anhui Province (KJ2020ZD70).

## Conflict of interest

The authors declare that the research was conducted in the absence of any commercial or financial relationships that could be construed as a potential conflict of interest.

## Publisher's note

All claims expressed in this article are solely those of the authors and do not necessarily represent those of their affiliated organizations, or those of the publisher, the editors and the reviewers. Any product that may be evaluated in this article, or claim that may be made by its manufacturer, is not guaranteed or endorsed by the publisher.
