# Supplementary material for: Patterns of adverse childhood experiences and depressive symptom trajectories in young adults: A longitudinal study of college students in China
Source: Front Psychiatry. 2022 Jul 25;13:918092. doi: 10.3389/fpsyt.2022.918092 (PMC9358020; doi:10.3389/fpsyt.2022.918092)
Supplement: Supplementary file 1 [file Data_Sheet_1.doc]

**Table DS 1 Indicators of fit for models with one through nine latent classes for ACEs**

| Class | No. of Free Parameters | AIC | BIC | aBIC | Entropy | LMR | BLRT | Proportions |
| --- | --- | --- | --- | --- | --- | --- | --- | --- |
| 1 | 21 | 63558.837 | 63689.158 | 63622.431 |  |  |  |  |
| 2 | 43 | 58908.210 | 59175.058 | 59038.426 | 0.740 | <0.001 | <0.001 | 0.612/0.389 |
| 3 | 65 | 56189.879 | 56593.254 | 56386.716 | 0.834 | <0.001 | <0.001 | 0.256/0.481/0.262 |
| 4 | 87 | 55504.968 | 56044.869 | 55768.426 | 0.866 | <0.001 | <0.001 | 0.064/0.258/0.469/0.209 |
| **5** | **109** | **54809.300** | **55485.728** | **55139.380** | **0.833** | **<0.001** | **<0.001** | **0.253/0.278/0.068/0.262/0.139** |
| 6 | 131 | 54298.929 | 55111.884 | 54695.631 | 0.823 | <0.001 | <0.001 | 0.260/0.277/0.069/0.169/0.119/0.106 |
| 7 | 153 | 53832.639 | 54782.121 | 54295.962 | 0.824 | <0.001 | <0.001 | 0.066/0.066/0.220/0.093/0.117/0.177/0.261 |
| 8 | 175 | 53663.566 | 54749.575 | 54193.511 | 0.829 | 0.0158 | <0.001 | 0.173/0.217/0.066/0.064/0.046/0.066/0.259/0.110 |
| 9 | 197 | 53532.905 | 54755.441 | 54129.472 | 0.828 | 0.4438 | <0.001 | 0.064/0.053/0.066/0.234/0.042/0.172/0.058/0.247/0.064 |

ACEs = Adverse Childhood Experiences;

AIC = Akaike’s Information Criteria;

BIC = Bayesian Information Criteria;

aBIC = sample size adjusted BIC;

LMRT = Lo-Mendell-Rubin Test

BLRT = Likelihood Ratio Test

**Table DS 2 Average Latent Class Probabilities for Most Likely Latent Class Membership (Row) by Latent Class (Column) for ACEs patterns**

ACEs = Adverse Childhood Experiences

|  | Class 1 | Class 2 | Class 3 | Class 4 | Class 5 |
| --- | --- | --- | --- | --- | --- |
| Class 1 High ACEs | **0.870** | 0.086 | 0.027 | 0.008 | 0.009 |
| Class 2 High neglect and emotional abuse | 0.051 | **0.843** | 0.006 | 0.087 | 0.014 |
| Class 3 High family dysfunction | 0.056 | 0.018 | **0.896** | 0.021 | 0.009 |
| Class 4 High neglect | 0.010 | 0.068 | 0.003 | **0.902** | 0.016 |
| Class 5 Low ACEs | 0.010 | 0.019 | 0.002 | 0.014 | **0.956** |

**Table DS 3 Indicators of fit for models with one through nine latent classes for ACEs (sensitivity analyses)**

| class | k | AIC | BIC | aBIC | Entropy | LMR | BLRT | Proportions |
| --- | --- | --- | --- | --- | --- | --- | --- | --- |
| 1 | 21 | 32160.394 | 32290.715 | 32223.987 |  |  |  | 1 |
| 2 | 43 | 24783.615 | 25050.463 | 24913.83 | 0.982 | <0.001 | <0.001 | 0.433/0.567 |
| 3 | 65 | 22645.516 | 23048.891 | 22842.353 | 0.978 | <0.001 | <0.001 | 0.382/0.535/0.082 |
| 4 | 87 | 21864.11 | 22404.012 | 22127.568 | 0.970 | <0.001 | <0.001 | 0.074/0.509/0.351/0.066 |
| **5** | **109** | **21347.643** | **22024.072** | **21677.723** | **0.952** | **<0.001** | **<0.001** | **0.072/0.054/0.058/0.334/0.481** |
| 6 | 131 | 20903.346 | 21716.301 | 21300.047 | 0.965 | <0.001 | <0.001 | 0.335/0.043/0.053/0.488/0.035/0.046 |
| 7 | 153 | 20577.552 | 21527.034 | 21040.876 | 0.975 | <0.001 | <0.001 | 0.489/0.047/0.036/0.044/0.338/0.028/0.018 |
| 8 | 175 | 20443.691 | 21529.699 | 21973.635 | 0.975 | 0.0151 | <0.001 | 0.045/0.047/0.032/0.012/0.010/0.338/0.030/0.486 |
| 9 | 197 | 20404.016 | 21626.551 | 21000.582 | 0.974 | 0.3535 | <0.001 | 0.007/0.009/0.032/0.007/0.044/0.337/0.486/0.049/0.029 |

ACEs = Adverse Childhood Experiences;

AIC = Akaike’s Information Criteria;

BIC = Bayesian Information Criteria;

aBIC = sample size adjusted BIC;

LMRT = Lo-Mendell-Rubin Test

BLRT = Likelihood Ratio Test


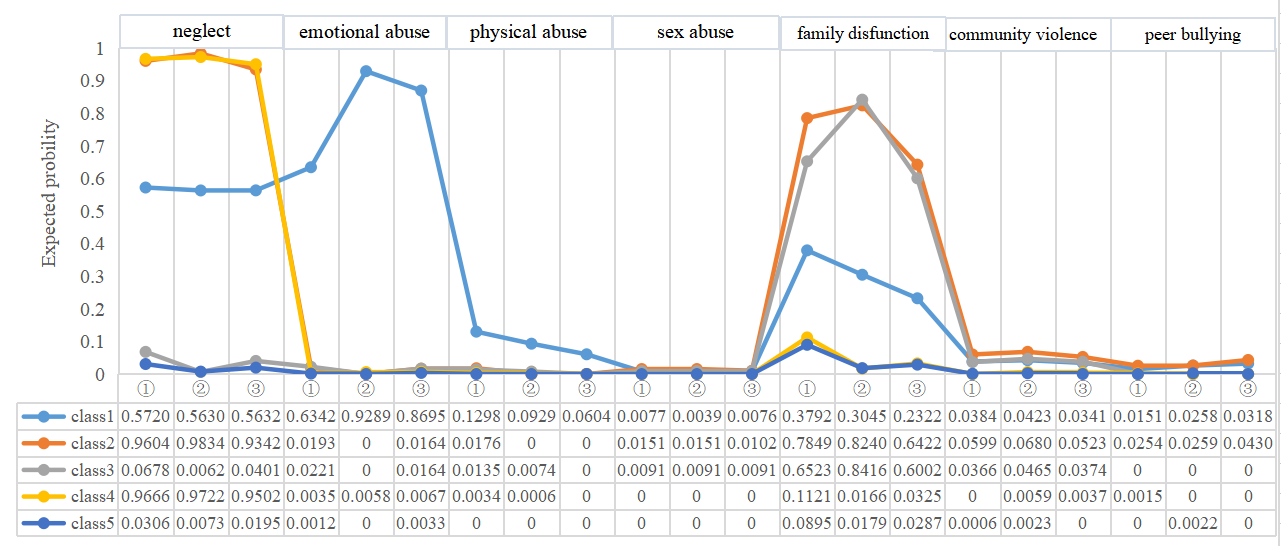


**Figure DS 1** Plot of 5 latent classes of adverse childhood experiences (sensitivity analyses)

(① = Before middle school; ② = In middle school; ③ = In high school )

**Table DS 4 Indicators of fit for models with one through four latent classes for depressive symptom**

| Class | No. of Free Parameters | AIC | BIC | aBIC | Entropy | LMR | BLRT |
| --- | --- | --- | --- | --- | --- | --- | --- |
| 1 | 5 | 77800.910 | 77831.938 | 77816.051 |  |  |  |
| 2 | 8 | 75188.453 | 75238.099 | 75212.679 | 0.869 | <0.001 | <0.001 |
| **3** | **11** | **74551.798** | **74620.061** | **74585.109** | **0.806** | **<0.001** | **<0.001** |
| 4 | 14 | 74283.831 | 74370.712 | 74326.227 | 0.829 | 0.1439 | <0.001 |

AIC = Akaike’s Information Criteria;

BIC = Bayesian Information Criteria;

aBIC = sample size adjusted BIC;

LMRT = Lo-Mendell-Rubin Test

BLRT = Likelihood Ratio Test

**Table DS 5 Average Latent Class Probabilities for Most Likely Latent Class Membership (Row) by Latent Class (Column) for depression trajectories.**

|  | **Class1** | **Class2** | **Class3** |
| --- | --- | --- | --- |
| **Class1:**High depressive symptom | **0.905** | 0.095 | 0.000 |
| **Class2:**Moderate depressive symptomn | 0.018 | **0.831** | 0.151 |
| **Class3:** Low depressive symptom | 0.000 | 0.095 | **0.905** |
